# Effects of Di-(2-ethylhexyl) Phthalate on Lipid Metabolism by the JAK/STAT Pathway in Rats

**DOI:** 10.3390/ijerph13111085

**Published:** 2016-11-04

**Authors:** Yiyang Jia, Te Liu, Liting Zhou, Jian Zhu, Juan Wu, Di Sun, Jin Xu, Qi Wang, Huaiji Chen, Feng Xu, Yuezhu Zhang, Tianrong Zhang, Hongbo Liu, Lin Ye

**Affiliations:** 1 Department of Occupational and Environmental Health, School of Public Health, Jilin University, Changchun 130021, China; jiayy14@mails.jlu.edu.cn (Y.J.); iamliute@126.com (T.L.); zhoulttg@163.com (L.Z.); zhujian@mails.jlu.edu.cn (J.Z.); s184349549@live.com (D.S.); xujin14@mails.jlu.edu.cn (J.X.); wangqi15@mails.jlu.edu.cn (Q.W.); chenhj15@mails.jlu.edu.cn (H.C.); xufeng15@mails.jlu.edu.cn (F.X.); s418824079@126.com (Y.Z.); zhangtr16@mails.jlu.edu.cn (T.Z.); liuhb@mails.jlu.edu.cn (H.L.); 2 Scientific Research Center, China-Japan Union Hospital, Jilin University, Changchun 130033, China; 3 Cancer Center, Tumor Hospital of Jiangxi Province, Nanchang 330029, China; wujuan13@mails.jlu.edu.cn

**Keywords:** di-(2-ethylhexyl) phthalate, JAK/STAT, lipid metabolism, adipose tissue

## Abstract

The most widely used plasticizer, di-(2-ethylhexyl) phthalate (DEHP), is known to affect lipid metabolism and adipogenesis. We studied the effects of dietary DEHP exposure on metabolism in rats as well as the role of the JAK/STAT pathway in this process. Eighty rats were exposed to DEHP (0, 5, 50 and 500 mg/kg/d) through dietary intake for 4 weeks. We then collected blood samples, liver, and adipose tissues to detect modifications in the levels of serum lipids, leptin, adiponectin and insulin. JAK3, STAT5a and PPARγ expression were detected at both the gene and protein levels. The activation of JAK3 and STAT5a was also detected. The DEHP-exposed rats had increased body weight, serum lipid, insulin, and leptin levels. Moreover, the JAK3/STAT5a pathway was activated in the adipose tissue; however, this pathway was not activated in the liver. The mRNA of SREBP-1c in the liver was increased significantly among each of the groups, in contrast to the levels found in the mature SREBP-1c protein form. Furthermore, the expression of *FABP4*, *Acox* and *FASn* was decreased in the liver, but increased in adipose tissue. Thus, we conclude that exposure to DEHP reduces the hydrolysis of lipid and promotes triglyceride accumulation by oppositely regulating the activation state of JAK/STAT pathway in the liver and adipose tissue, resulting in the disorder of body lipid metabolism and obesity.

## 1. Introduction

The most widely used plasticizer, di-(2-ethylhexyl) phthalate (DEHP), is known to cause reproductive toxicity and possesses endocrine disruptive potential [1,2]. This synthetic chemical is known to be an endocrine-disrupting chemical, which are known to adversely affect the endocrine system, and can be found in a variety of plastic products, household paints, as well as toys used by children. Human exposure to DEHP is reported to be mainly through ingestion and dermal contact [3]. In recent years, a number of studies have shown that exposure to DEHP can cause abnormal lipid metabolism in humans, which results in numerous health implications such as heart disease, diabetes, and as the most common disorder, obesity [4,5,6].

Compared with adults, children have increased activity and exposure to chemicals due to objects touched with their hands and placed in their mouths. Thus, children are more likely to be exposed to phthalates. Moreover, several studies suggest that DEHP may promote childhood obesity [7]. Hou’s study suggests that DEHP exposure is associated with abdominal obesity in adolescents [8]. Kim et al. found that DEHP exposure may have a close relationship with the incidence of obesity in school-age children [9]. 

Reports show that DEHP promotes the expression of transcriptional factors such as peroxisome proliferator-activated receptor γ (PPARγ), sterol regulatory element binding proteins (SREBP) as well as downstream target genes that are required for adipogenesis in vivo. PPARγ, a nuclear hormone receptor, is mainly expressed in adipose tissue and plays a number of key roles including regulating the differentiation of adipocytes and fat accumulation/storage in the adipose tissue. Mono-(2-ethylhexyl) phthalate (MEHP), which is a metabolite of DEHP, can act as a PPARγ agonist, thereby promoting differentiation and lipid accumulation in 3T3-L1 cells [8,10]. However, Hao et al. observed that MEHP activated PPARγ and its target genes in vivo [11]. Therefore, it is likely that phthalates exert an adipogenic effect through the activation of PPARγ [12]. 

Studies have shown that lipid metabolism is related to the JAK/STAT signal transduction pathway [13,14]. In a study of lipid metabolism involving flies, it was reported that a lipid-rich diet contributed to weight gain, insulin resistance and hyperglycemia, as well as systemic activation of the JAK/STAT signaling pathway [15]. STATs and PPARγ, the major adipogenic transcription factors, can be regulated by each other. Furthermore, DEHP regulates the expression of specific transcription factors through the JAK/STAT signal transduction pathway, and may have an effect on lipid metabolism.

Taken together, we sought to expose younger rats (age 3 weeks) to DEHP until reaching adolescence to assess the toxic effect of DEHP on growth and development. In the study herein, we hypothesized that exposure to DEHP was related to lipid metabolism disorder, and that is mediated by the JAK/STAT pathway. We aimed, using a rat model, to evaluate the effects of DEHP on lipid metabolism, and to identify whether the JAK/STAT pathway plays a role in this process. The exposure dose was set from the no observed adverse effect level (NOAEL) of DEHP (5 mg/kg/d) [16]—up to the high DEHP dose level (500 mg/kg/d).

## 2. Materials and Methods

### 2.1. Preparations and Animals

All experimental animals were purchased from the Experimental Animal Center of Jilin University (Changchun, China). Prior to initiating the study, the experimental protocol was subject to approval by the Animal Use and Care Committee of Jilin University (Code: 130021). Corn oil (Aladdin, Shanghai, China) was used to dissolve DEHP (Sinopharm Chemical Reagent Co., Shanghai, China, purity > 99%) at different concentrations.

A total of eighty Wistar weaning rats (age: 3 weeks, 60 ± 10 g, half male and half female) were randomly divided into four groups and exposed to different concentrations of dietary DEHP (0, 5, 50, and 500 mg/kg/d) by gavage for a period of 4 weeks. Food intake and weight gain were measured each day. The rats were housed individually under controlled light and temperature conditions (12 h light/dark cycle; 23 ± 1 °C) with free access to food and water.

### 2.2. Tissue Collection

After 4 weeks, the rats were weighed and anesthetized by 3.5% chloral hydrate in asepsis condition. Blood samples were drawn from the heart of each rat using vacuum blood collection tubes. The serum specimens were centrifuged at 2500 rpm for 20 min and then the sera were separated and stored at −20 °C. Following the blood collection, liver and perianal fat were isolated and maintained in liquid nitrogen until RNA extraction.

### 2.3. Detection of Lipid and Hormone in Serum

According to the instructions provided by the manufacturer, the levels of triglycerides (TG), high density lipoprotein (HDL), low density lipoprotein (LDL), total cholesterol (CHO), insulin, leptin and adiponectin in the serum were assayed using an ELISA kit (R&D Systems, Minneapolis, MN, USA).

### 2.4. RNA Extraction and RT-PCR Analysis

We used Trizol reagent (TaKaRa, Kusatsu, Shiga, Japan) to extract total RNA from liver and perirenal fat tissue. The amount of total RNA was determined by spectrophotometry and RNA purity was determined by the 260/280 nm ratio. The ratio values greater than 1.8 means RNA quality accords to the standard.

A real-time quantitative PCR method using a Stratagene MX3000p device (Stratagene, La Jolla, CA, USA) was used to assess the expression of genes, and Table 1 shows the primer sequences used for amplification of each gene. SYBR Premix Ex Taq II (TaKaRa) was used according to the manufacturer’s instructions, the PCR reaction was carried out in 45 cycles of 95 °C for 20 s and 60 °C for 20 s. For each sample, q RT-PCR was run in triplicate to ensure consistency. The relative expression levels were determined by normalizing to β-actin, the housekeeping gene.

### 2.5. Immunohistochemistry

To conduct the immunohistochemistry (IHC), tissues were fixed in 10% formalin and embedded in paraffin, and then cut into 5-µm thick slides. The sections were sequentially submerged in xylene and gradually increasing concentrations of ethanol to dewax. Endogenous peroxidase activity was blocked with 10% H_2_O_2_ for 10 min at room temperature. Unspecific binding was blocked using rat serum at a dilution of 1:10 for 30 min at room temperature. Then, adipose tissue sections were incubated with anti-rabbit STAT5a (Abcam, Cambridge, UK), anti-mouse JAK3 (Santa Cruz Biotechnology, Santa Cruz, CA, USA), and anti-mouse PPARγ (Abcam). In addition to these antibodies, liver sections were incubated with anti-rabbit SREBP-1c (Abcam) as the primary antibody. After washing three times for 5 min using 1× PBS, each section was incubated with the appropriate secondary antibody for 30 min, followed by streptavidin—peroxidase complex for 10 min at room temperature.

### 2.6. Western Blot

Determinations of JAK3, STAT5a, PPARγ and SREBP-1c were assessed using western blot. Proteins were extracted from liver and adipose tissue. The concentration of protein samples was determined using the bicinchoninic acid protein assay kit (Beyotime, Shanghai, China). The proteins were separated by SDS-PAGE (Beyotime) and transferred to a nitrocellulose membrane (Pall, Ann Arbor, MI, USA). The membrane was then incubated in blocking solution with anti-JAK3 (Santa Cruz Biotechnology), anti-STAT5a (Abcam), anti-PPARγ (Abcam) and anti-SREBP-1c (Abcam), respectively. The phosphorylated forms of JAK3 and STAT5a were detected using anti-STAT5a (phospho S726, Abcam) and anti-phospho-JAK3 (pTyr785, Sigma-Aldrich, St. Louis, MO, USA). Following the incubation of the secondary antibodies, enhanced chemiluminescence substrate (Beyotime) was then applied for 5 min prior to exposure. The activated state of JAK3 and STAT5a is displayed as the ratio of phosphorylated protein/total protein.

### 2.7. Statistical Analysis

All data were tested by normality (Kolmogorov-Smirnov test) and homogeneity of variance (Levene’s test). Conditional data are presented as mean ± standard error of mean (SE). We performed one-way ANOVA on each parameter followed by a Tukey’s post hoc test at each sampling time separately. The Mann-Whitney rank-sum test was employed for the nonparametric analysis. Box diagrams were used to describe the PCR and ELISA results, where the maximum, minimum and median values were displayed. Differences between groups were considered statistically significant if *p* < 0.05.

## 3. Results

### 3.1. Effects of 4 Weeks of Dietary Exposure to DEHP on Body Weight in Rats

**Figure 1:**
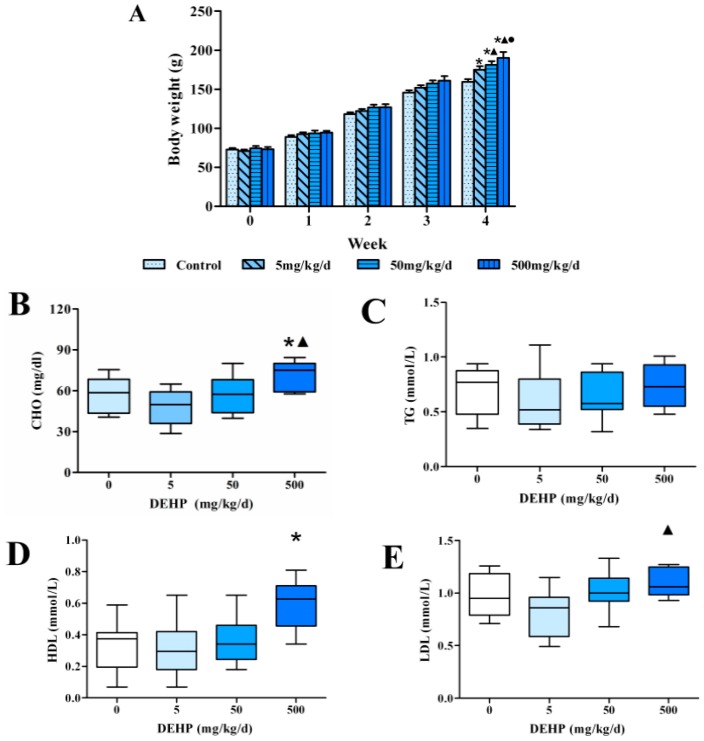

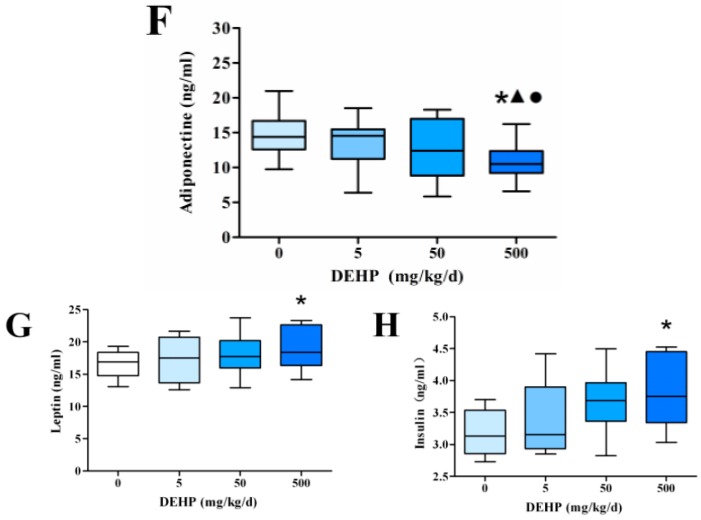
Effects of 4 weeks of dietary exposure to DEHP on body weight, serum lipid levels and related hormone levels in rats (*n* = 20 animals/ each group). (**A**) The body weights; (**B**) Total cholesterol (CHO) level; (**C**) Triglyceride (TG) level; (**D**) High density lipoprotein (HDL) level; (**E**) Low density lipoprotein (LDL) level; (**F**) Adiponectin level; (**G**) Leptin level; (**H**) Insulin level. The body weight was expressed as the mean value ± standard error (SE), serum lipid levels and related hormone levels were expressed as the min to max and the median. ● Statistically significant difference compared with 50 mg·kg^−1^·d^−1^ (*p* < 0.05); ▲ Statistically significant difference compared with 5 mg·kg^−1^·d^−1^ (*p* < 0.05); * Statistically significant difference compared with control (*p* < 0.05).

Although an upward trend in body weight was displayed in each of the rats, the body weight of the rats that were exposed to DEHP was higher compared to the control group. Before experiment, the average body weight in control group was 72.65 g, in 5 mg/kg/d group was 70.65 g, in 50 mg/kg/d group was 74.2 g and in 500 mg/kg/d group was 73.05 g. After 4 weeks of exposure to DEHP, we observed statistically significant differences among all of the groups analyzed in this study (All *p* < 0.05, Figure 1A). The final average body weight in control group was 159.8 g, which was 2.2 times than that before exposure. In 5 mg/kg/d group, the final average body weight was 174.8 g, which was 2.47 times than that before exposure. In 50 mg/kg/d group, the final average body weight was 181.2 g, which was 2.44 times than that before exposure. In 500 mg/kg/d group, the final average body weight was 190.2 g, which was 2.60 times than that before exposure. The results showed a linear effect in that the average weight of the rats increased with the exposure dose.

### 3.2. Effect of DEHP on Serum Lipid Levels in Rats

We detected the levels of lipids in serum of rats and found that there was no statistically significant difference in the content of serum TG of the rats in each group (*p* < 0.05, Figure 1C). The serum CHO level in 500 mg/kg/d group was significantly higher than that in 5 mg/kg/d group and control (*p* < 0.05, Figure 1B). However, the level of serum HDL in 500 mg/kg/d group was significantly higher than the control (*p* < 0.05, Figure 1D). Moreover, the serum levels of LDL in 500 mg/kg/d group were significantly higher than that in 5 mg/kg/d group, and the difference was statistically significant (*p* < 0.05, Figure 1E).

### 3.3. Effect of DEHP on Serum Lipid Metabolism Related Hormone Levels in Rats

Compared with the control group, the levels of serum leptin (Figure 1G) and insulin (Figure 1H) were significantly higher in the 500 mg/kg/d group, but the level of adiponectin (Figure 1F) was significantly decreased (All *p* < 0.05). The level of serum insulin may be increased by insulin-resistance, which means that their cells are not responsive to insulin. Consistent with this result is the reduction in serum adiponectin, which is in good agreement with the increase in leptin expression. The decrease of plasma adiponectin level was associated with the increase of body mass index, decreased insulin sensitivity, and increased risk of cardiovascular disease.

### 3.4. Gene Expression Analysis in the Liver

**Figure 2:**
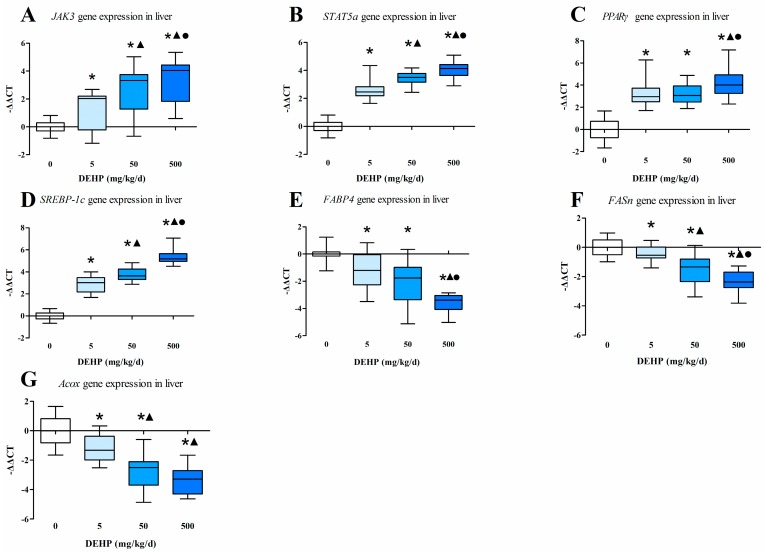
Effects of DEHP on expression of the JAK3/STAT5 pathway genes in liver (*n* = 20 animals/each group): (**A**) The fold change of mRNA levels of *JAK3* in liver; (**B**) The fold change of mRNA levels of *STAT5a* in liver; (**C**) The fold change of mRNA levels of *PPARγ* in liver; (**D**) The fold change of mRNA levels of *SREBP-1c* in liver; (**E**) The fold change of mRNA levels of *FABP4* in liver; (**F**) The fold change of mRNA levels of *FASn* in liver; (**G**) The fold change of mRNA levels of *Acox* in liver. All data was expressed as the min to max and the median. ● Statistically significant difference compared with 50 mg·kg^−1^·d^−1^ (*p* < 0.05); ▲ Statistically significant difference compared with 5 mg·kg^−1^·d^−1^ (*p* < 0.05); * Statistically significant difference compared with control (*p* < 0.05).

We found that in the liver the mRNA levels of *JAK3* (Figure 2A), *STAT5a* (Figure 2B) and *SREBP-1c* (Figure 2D) were different among all groups. The mRNA levels of *PPARγ* (Figure 2C) in the DEHP-treat groups were all significantly higher than the control, and in the 500 mg/kg/d group the *PPARγ* level was significantly higher compared with other groups (All *p* < 0.05).

In regards to the downstream genes, the mRNA levels of fatty acid binding protein fatty 4 (*FABP4*, also known as *aP2*) (Figure 2E), fatty acid synthetase (*FASn*, also known as *FAS*) (Figure 2F) and acyl CoA oxidase (*Acox*, also known as *AOX* or *ACO*) (Figure 2G) in the DEHP-treated groups were lower than the mRNA levels observed in the control group (All *p* < 0.05). 

### 3.5. Gene Expression Analysis in Fat Tissue

**Figure 3:**
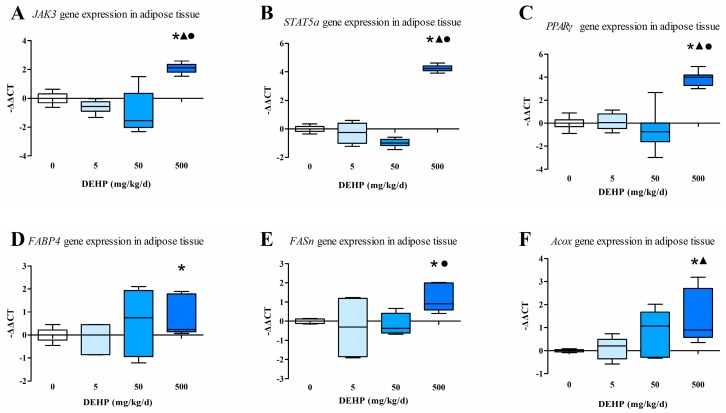
Effects of DEHP on expression of the JAK3/STAT5 pathway genes in adipose tissue (*n* = 20 animals/ each group): (**A**) The fold change of mRNA levels of *JAK3*; (**B**) The fold change of mRNA levels of *STAT5a*; (**C**) The fold change of mRNA levels of *PPARγ*; (**D**) The fold change of mRNA levels of *FABP4*; (**E**) The fold change of mRNA levels of *FASn*; (**F**) The fold change of mRNA levels of *Acox*. All data was expressed as the min to max and the median. ● Statistically significant difference compared with 50 mg·kg^−1^·d^−1^ (*p* < 0.05); ▲ Statistically significant difference compared with 5 mg·kg^−1^·d^−1^ (*p* < 0.05); * Statistically significant difference compared with control (*p* < 0.05).

In the fat tissues, we found that the mRNA levels of *JAK3* (Figure 3A), *STAT5a* (Figure 3B), *PPARγ* (Figure 3C), of the group exposed to 500 mg/kg/d of DEHP were significantly higher than those observed in other groups (All *p* < 0.05). The mRNA level of *FASn* (Figure 3E) in 500 mg/kg/d group was significantly higher than the 50 mg/kg/d group and the control. The mRNA level of *Acox* (Figure 3F) in 500 mg/kg/d group was significantly higher than the 5 mg/kg/d group and the control. The mRNA level of *FABP4* was significantly higher in the group exposed to 500 mg/kg/d DEHP compared with the control group (*p* < 0.05, Figure 3D).

### 3.6. Protein Expression in the Liver

**Figure 4:**
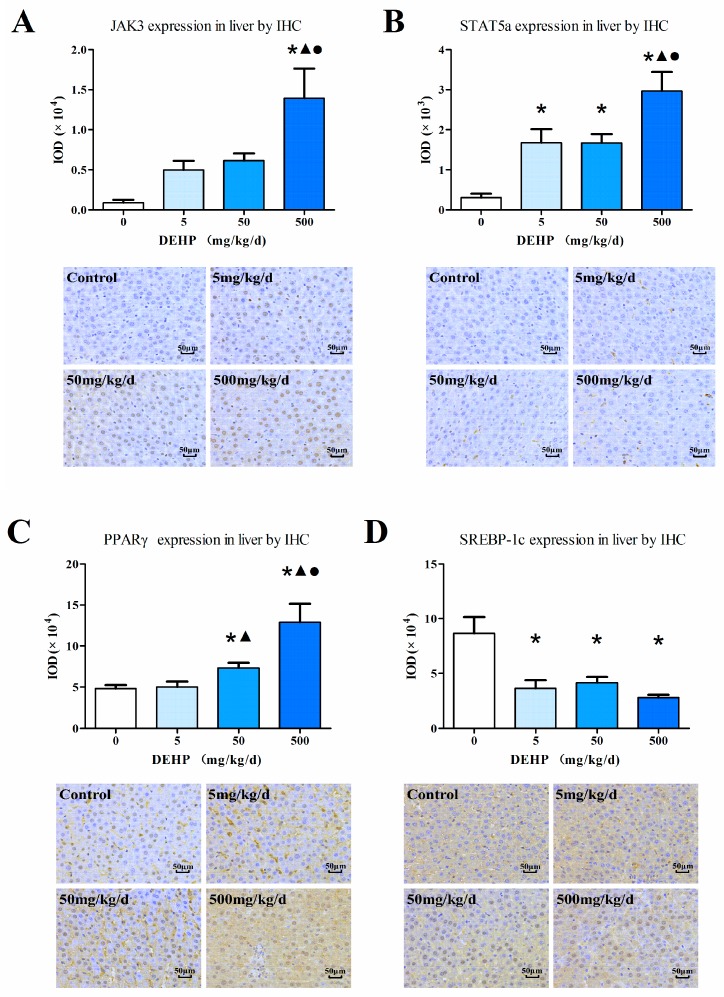
Immunohistochemical determination of proteins in the liver (*n* = 20 animals/each group): (**A**) Immunohistochemical staining of JAK3 expression in the liver (400×); (**B**) Immunohistochemical staining of STAT5a expression in the liver (400×); (**C**) Immunohistochemical staining of PPARγ expression in the liver (400×); (**D**) Immunohistochemical staining of SREBP-1c expression in the liver (400×); Brown staining indicates areas of positive protein expression. Results were expressed as the mean ± SE (*n* = 20 animals/treatment group). ● Statistically significant difference compared with 50 mg·kg^−1^·d^−1^ (*p* < 0.05); ▲ Statistically significant difference compared with 5 mg·kg^−1^·d^−1^ (*p* < 0.05); * Statistically significant difference compared with control (*p* < 0.05).

The intensity of the immunohistochemical protein staining differs among the groups examined in this study (Figure 4). The results showed increased protein expressions of JAK3 (Figure 4A), STAT5a (Figure 4B) and PPARγ (Figure 4C) in the liver of the group exposed to 500 mg/kg/d of DEHP compared with the other groups (All *p* < 0.05). Furthermore, the protein expression of SREBP-1c was significantly decreased in the DEHP-treated groups compared with the control (*p* < 0.05, Figure 4D).

**Figure 5:**
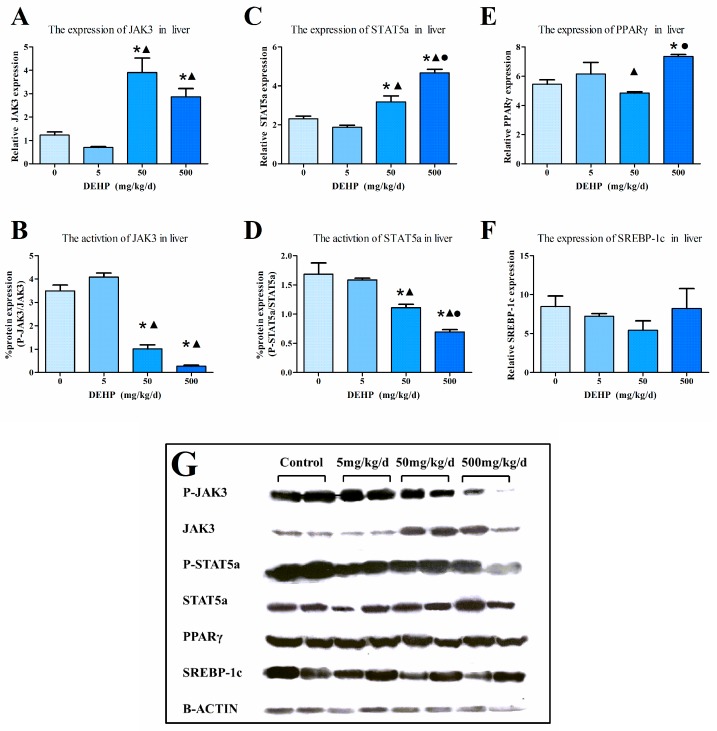
Western blot determination of proteins in the liver (*n* = 20 animals/ each group): (**A**) The densitometric scans of JAK3/β-actin; (**B**) The densitometric scans of P-JAK3/JAK3; (**C**) The densitometric scans of STAT5a/β-actin; (**D**) The densitometric scans of P-STAT5a/STAT5a; (**E**) The densitometric scans of PPARγ/β-actin; (**F**) The densitometric scans of SREBP-1c/β-actin; (**G**) Western blot assay of JAK3, P-JAK3, STAT5, P-STAT5a, PPARγ and SREBP-1c expression in the liver after DEHP treatment. The histogram represents the mean ± SE of the densitometric scans for protein bands from each group and normalized to β-actin. ● Statistically significant difference compared with 50 mg·kg^−1^·d^−1^ (*p* < 0.05); ▲ Statistically significant difference compared with 5 mg·kg^−1^·d^−1^ (*p* < 0.05); * Statistically significant difference compared with control (*p* < 0.05).

To confirm the results from the immunohistochemical analysis, we conducted western blots and found that the results from the two analyses to be mostly consistent with one another (Figure 5G). The protein expressions of JAK3 (Figure 5A), STAT5a (Figure 5C) and PPARγ (Figure 5E) of the high DEHP dose group were significantly higher than the control group (All *p* < 0.05). However, there was no statistically significant difference in the SREBP-1c levels in all groups (*p* > 0.05, Figure 5F). We further examined the phosphorylated state of the proteins and found that the activated state of JAK3 (Figure 5B) in the 50 mg/kg/d and 500 mg/kg/d DEHP group were significantly lower than activated state observed in the 5 mg/kg/d DEHP and control group (*p* < 0.05). There were no statistically significant differences among the activated state of STAT5a (Figure 5D) in the 5 mg/kg/d DEHP group and the control group (*p* < 0.05). Furthermore, our findings showed decreased activated STAT5a protein in the 50 mg/kg/d DEHP and 500 mg/kg/d DEHP group compared with the 5 mg/kg/d DEHP group and the control group. 

### 3.7. Protein Expression in Fat Tissue

**Figure 6:**
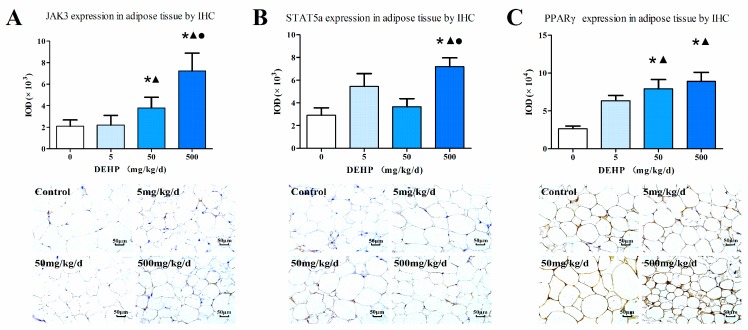
Immunohistochemical determination of proteins in the adipose tissue (*n* = 20 animals/each group): (**A**) Immunohistochemical staining of JAK3 expression in the adipose tissue (400×); (**B**) Immunohistochemical staining of STAT5a expression in the adipose tissue (400×); (**C**) Immunohistochemical staining of PPARγ expression in the adipose tissue (400×); Brown staining indicates areas of positive protein expression. Results were expressed as the mean ± SE (*n* = 20 animals/treatment group). ● Statistically significant difference compared with 50 mg·kg^−1^·d^−1^ (*p* < 0.05); ▲ Statistically significant difference compared with 5 mg·kg^−1^·d^−1^ (*p* < 0.05); * Statistically significant difference compared with control (*p* < 0.05).

Immunohistochemical analysis (Figure 6) showed an increased protein expression of JAK3 as the dose of DEHP increased; however, the protein level of JAK3 in the 5 mg/kg/d DEHP group displayed no statistically significant difference compared with the control group (*p* < 0.05, Figure 6A). Compared with other groups, the 500 mg/kg/d DEHP group displayed a higher expression of STAT5a (*p* < 0.05, Figure 6B). In regards to the protein levels of PPARγ, the 500 and 50 mg/kg/d groups were statistically significant than the 5 mg/kg/d group and the control (*p* < 0.05, Figure 6C).

**Figure 7:**
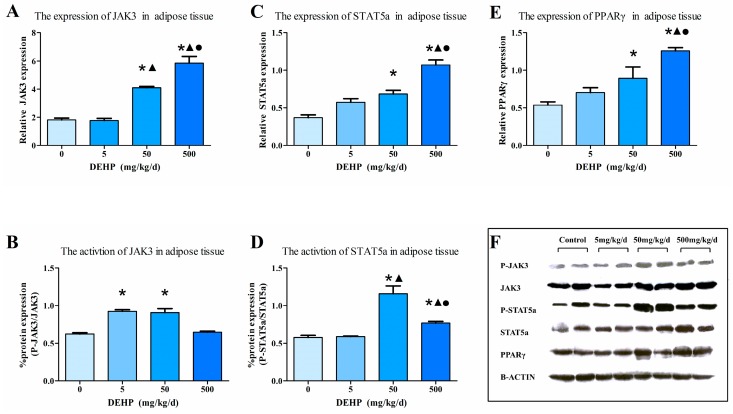
Western blot determination of proteins in the adipose tissue (*n* = 20 animals/ each group): (**A**) The densitometric scans of JAK3/β-actin; (**B**) The densitometric scans of P-JAK3/JAK3; (**C**) The densitometric scans of STAT5/β-actin; (**D**) The densitometric scans of P-STAT5a/STAT5a; (**E**) The densitometric scans of PPARγ/β-actin; (**F**) Western blot assay of JAK3, P-JAK3, STAT5a, P-STAT5a and PPARγ expression in the adipose tissue after DEHP treatment. The histogram represents the mean ± SE of the densitometric scans for protein bands from each group and normalized to β-actin. ● Statistically significant difference compared with 50 mg·kg^−1^·d^−1^ (*p* < 0.05); ▲ Statistically significant difference compared with 5 mg·kg^−1^·d^−1^ (*p* < 0.05); * Statistically significant difference compared with control (*p* < 0.05).

Western blot analysis (Figure 7F) showed that the protein expression of JAK3 (Figure 7A), STAT5a (Figure 7C) and PPARγ (Figure 7E) of the 500 mg/kg/d DEHP group was significantly increased compared with other groups. The expression levels of these three proteins in the 50 mg/kg/d DEHP group were significantly higher than that in the control group (All *p* < 0.05). The activation of JAK3 (Figure 7B) protein in the 5 mg/kg/d and 50 mg/kg/d DEHP group was found to be up-regulated compared with the control group (*p* < 0.05). Furthermore, the activation of STAT5a (Figure 7D) protein in the 50 mg/kg/d and 500 mg/kg/d DEHP group was up-regulated compared with the 5 mg/kg/d DEHP group and control group. In the 500 mg/kg/d DEHP group, the activation of STAT5a protein was significantly lower than that observed in the 50 mg/kg/d DEHP group (*p* < 0.05).

## 4. Discussion

Lipid metabolism disorder is a global public health problem attributable to a complex interaction between genetic, behavioral, and environmental factors. However, some scholars have identified that exposure to environmental chemicals during the developmental stages may be a contributing factor to the obesity epidemic [17,18,19]. Current research of JAK3/STAT5 signaling has focused on its role in the immune system and the occurrence and development of various cancers [20,21,22]. Limited studies exist regarding JAK expression, activation and function in fat cells and adipose tissue. In contrast, STATs have been well studied and documented regarding their role in adipocytes [23]. In vivo, lipid metabolism has two stages: adipogenesis and lipid accumulation. Previous studies have shown that the JAK/STAT pathway plays a different role in the different stages of lipid metabolism [14,24,25].

In the liver, our present data showed a dose-response relationship between the dose and the mRNA levels of *JAK3* and *STAT5a* in which statistically significant differences exist between each group. In contrast, the dose-response relationship was not observed in the adipose tissue, where we only detected significantly increased mRNA levels of *JAK3* and *STAT5a* in the 500 mg/kg/d DEHP group. These results suggest that the gene expression of *JAK3* and *STAT5a* is up-regulated after exposure to DEHP; however, the regulation of DEHP dose varies among different tissues. 

Next, we sought to detect protein levels using IHC and western blot analyses. Compared with the control, the expression of JAK3 protein in the 500 mg/kg/d DEHP group and the expression of STAT5a protein in all of the DEHP-exposed groups were significantly increased in the liver. In adipose tissue, JAK3 and STAT5a protein expression was up-regulated in the 50 mg/kg/d and 500 mg/kg/d DEHP group. In consideration that the mRNA levels of JAK3 and STAT5a were not significantly increased in the 50 mg/kg/d DEHP group, we hypothesized that DEHP exposure could promote the transcription and translation of JAK3 and STAT5a.

We next sought to determine the level of activation among JAK3 and STAT5a. The results showed that the activation states of JAK3 and STAT5a was inconsistent between the levels found in total protein and those found in various tissues. The activation of JAK3 and STAT5a in the 50 and 500 mg/kg/d DEHP group is highly decreased compared with the control group, indicating that the JAK3/STAT5 pathway was inhibited in the liver. On the contrary, we found that the JAK3/STAT5 pathway was activated in the adipose tissue. 

JAKs are largely controlled by tyrosine phosphorylation, rather than by expression levels. Currently, there is no evidence that JAKs play a STAT-independent role in modulating adipocyte differentiation in white adipose tissue [14,23]. However, a study that examined JAK3 knockout mice found that the loss of JAK3 leads to increased body-weight and impaired ability in glucose homeostasis [26]. Although tyrosine phosphorylation is critical for canonical STAT activation, other covalent modifications such as serine phosphorylation, acetylation, methylation and sumoylation can also occur. These remarkably proficient proteins also provide non-canonical functions in transcriptional regulation [27]. STAT5 proteins appear to have both adipogenic and anti-adipogenic effects in adipose tissue, which may be related to the developmental stage of the tissue [28]. Moreover, PPARγ is induced during differentiation of pre-adipocytes to adipocytes and is essential for this process [29]. Without PPARγ, precursor cells are unable to differentiate into mature adipocytes [30].

STAT5 proteins that are activated early and directly transactivate the PPARγ promoters in adipogenesis [31]. The PPARγ gene is a STAT5 target during the development of adipocytes and the protein may influence the role of STAT5 in adipogenesis. In the present study, the detection of the gene and protein levels of PPARγ showed that the expression of PPARγ was up-regulated in both the liver and adipose tissue. In adipose tissue, different results were obtained between the IHC and western blot methods. We speculate that these inconsistencies may be related to the idea that the IHC method is easily subjected to obtaining false-positive results. In adipose tissue, PPARγ can activate lipoprotein lipase, thereby limiting triglyceride decomposition, and promoting the maturity of fat cells through fatty acid uptake and storage [32]. PPARγ activation can accelerate the decomposition of triglycerides in the peripheral tissues, increase their synthesis in adipose tissue and inhibit the formation of glucagon [33]. In addition, activation of PPARγ can promote the removal of lipids and fatty acids in adipose tissue. In the liver, the different expression trends of PPARγ and phosphorylated STAT5a implied that the role of PPARγ in the JAK/STAT pathway may not occur downstream of the activated STAT5a. Mynatt et al. suggest that PPARγ can inhibit the JAK/STAT pathway in the leptin signal transduction pathway, thereby blocking the inhibitory effect of leptin on insulin secretion [34]. The increased expression of PPARγ in liver may be a compensatory response to weaken the insulin resistance. 

In the SREBPs family, SREBP-1c has a greater role in regulating genes associated with fatty acid synthesis. However, ectopic expression of a dominant-negative SREBP-1c has been shown to attenuate adipocyte differentiation [35]. In addition, SREBP-1c can up-regulate the function of PPARγ; however, the overexpression of SREBP-1c has been shown to enhance the adipogenic activity of PPARγ. Other studies suggest that SREBP-1c contributes to the generation of PPARγ ligands [36]. We analyzed the mRNA and protein levels of SREBP-1c in the liver and found that the mRNA levels were significantly increased as the dose of DEHP increased; however, this effect was not observed at the protein level. The IHC stains showed that the SREBP-1c protein in all of the DEHP-exposed groups were lower than that found in the control group, and this is consistent with a decrease in the transcriptional level of target genes (*FABP4*, *FASn* and *Acox*) in the liver. 

*FASn* is a key enzyme in fatty acid synthesis, and can significantly increase the deposition of TG in the body and lead to obesity. The promoter for *Acox*, is a rate limiting enzyme in peroxisomal fatty acid β-oxidation. The *Acox* gene contains a STAT5 binding site that modulates its gene expression in fat cells. Transfection studies showed that the promoter of *FABP4* was activated by STAT5. Interestingly, the expression of *FABP4* was inhibited by STAT5 found in the former fat cells of the mice examined. It has been shown that *FABP4* interacts with the non-phosphorylated form of JAKs [37]. We analyzed the mRNA levels of *FASn*, *FABP4* and *Acox* in the liver and found that the gene expression of these enzymes showed a downward trend, indicating that following DEHP exposure, the ability to synthesize fatty acids was decreased in the liver. Detection of mRNA levels of these enzymes in adipose tissue revealed a contrasting result within the liver. Compared with the control, the mRNA levels of *FASn*, *FABP4* and *Acox* were significantly increased in the 500 mg/kg/d DEHP group. This result suggested that following 500 mg/kg/d of exposure to DEHP, the ability of adipose tissue to synthesize and store fatty acids is increased, and this increased synthesis and storage will promote the accumulation of lipids. In the process of lipid metabolism, free fatty acids in the serum are transported to the liver and synthesized into TG, in turn, the adipose tissue stores these TG in the adipocytes. In contrast with the results of our studies, some studies have indicated that exposure to DEHP leads to an increase in lipid metabolism-related genes in both the liver and adipose tissue [38].

Cytokines and hormones are known to act through JAK/STAT signaling pathways. The hormone leptin, which plays a vital role in appetite regulation, signals through the JAK/STAT pathway. Leptin is synthesized by adipocytes and reflects the number and size of adipocytes in the body [39]. In the present study, the serum leptin levels were elevated with increasing doses of DEHP. In the 500 mg/kg/d DEHP group, the leptin level was significantly higher than the control group and this supports the hypothesis that DEHP exposure can lead to increased lipid accumulation in the body. While examining another hormone, adiponectin, we observed that the serum level in the 500 mg/kg/d DEHP group was higher than all other groups and the difference was statistically statistically significant. 

Many studies have found that there exists a statistically significant inverse relationship between adiponectin and the amount of fat [40,41]. The level of serum insulin may be increased due to increased insulin resistance, an indication that the cells are non-responsive to insulin. Consistent with this result is the reduction in serum adiponectin as observed in the 500 mg/kg/d group, which is in agreement with the increased leptin expression. 

In determining the levels of serum lipid, we found that only in the 500 mg/kg/d DEHP group, the levels of HDL was significantly higher than the control group. Moreover, the concentrations of TG were not significantly different among the examined groups. To clarify, at a relatively low dose (5 and 50 mg/kg/d), DEHP exposure did not appear to have a statistically significant effect on serum lipid concentrations. We speculate that this is related to the regulatory mechanisms inherent to the organism. The presence of high levels of blood lipids can inhibit the ability of the liver to synthesize lipids and promote adipose tissues to accelerate the storage of lipids [42]. Furthermore, these results provide an explanation regarding the reduction of lipid metabolism-related enzymes in the liver, and an increase of these enzymes in the adipose tissue.

In our study, the lowest DEHP dose (5 mg/kg/d) led to a statistically significant increase in body weight. In 2003, the World Health Organization issued that the tolerable DEHP daily intake in humans is 0.5 mg/kg, which is one tenth of our lowest dose. However, other researchers found that when exposed to 0.5 mg/kg/d DEHP, the C3H/N mice displayed weight gain and visceral fat increase [5]. In our study, in the adipose tissue, the change of JAK3, STAT5a, PPARγ and lipid metabolism-related enzymes was only observed in the 500 mg/kg/d group, which can lead to obvious reproductive toxicity in animals. In the 500 mg/kg/d group, we also observed more fat cells in the same section. Moreover, it may be difficult for human beings to receive such a large dose of DEHP exposure in their daily lives. Some indicators related to lipid metabolism, such as the serum lipids and hormones, were not significantly modified in the 5 mg/kg/d and 50 mg/kg/d DEHP group. Considering that adipose tissue is composed of a variety of cells, it is still not clear whether the capillary cells and connective tissue cells have an influence in the results. To expand upon the results of the present study, our future investigations will seek to elucidate the influence of DEHP on lipid metabolism through the JAK/STAT pathway in vitro. Furthermore, we will analyze other members of the JAK/STAT family, using various knockout models.

## 5. Conclusions

In this present study, we found that the DEHP-exposed rats displayed increased body weight, serum lipid, insulin and leptin levels, in addition to lower adiponectin levels. After exposure to DEHP, the mRNA and protein levels of JAK3 and STAT5a were increased in both the liver and adipose tissue; however the activation of JAK3 and STAT5a were inhibited in the liver and activated in the adipose tissue. In the liver, the downstream expression of lipogenic genes was decreased, thus reducing the ability of the liver to synthesize and conduct lipolysis. In contrast, the impact of the JAK/STAT pathway upon exposure to DEHP in the adipose tissue is reversed in that we observed increased levels of accumulating triglycerides. These results suggest that exposure to DEHP reduces the hydrolysis of lipid and promotes triglyceride accumulation by oppositely regulating the activation state of the JAK/STAT pathway in the liver and adipose tissue, resulting in the disorder of body lipid metabolism and obesity. 

## Figures and Tables

**Table 1:** Real time quantitative PCR primers.

Gene	Sequence 5′-3′	Sequence 3′-5′
*JAK3*	AGTGAGGCGCATGTGAAGATTG	CCGAAGCTCCACACGTCAGA
*STAT5a*	GTGCCCTCAGGCTCACTACAAC	CCACATGCCTGGCAACATC
*PPARγ*	GGAGCCTAAGTTTGAGTTTGCTGTG	TGCAGCAGGTTGTCTTGGATG
*SREBP-1c*	CCCTGCGAAGTGCTCACAA	GCGTTTCTACCACTTCAGGTTTCA
*FABP4*	CCTTTGTGGGGACCTGGAAA	TGACCGGATGACGACCAAGT
*FASn*	TGGTCACAGACGATGACAGGA	AGGCGTCGAACTTGGACAGA
*Acox1*	ATTGGCACCTACGCCCAGAC	CCAGGCCACCACTTAATGGAA
*β-actin*	CCCATTGAACACGGCATTG	GGTACGACCAGAGGCATACA

## Abbreviations

The following abbreviations are used in this manuscript:

DEHP	di-(2-ethylhexyl) phthalate
MEHP	Mono-(2-ethylhexyl) phthalate
JAK	Janus Kinase
STAT	Signal transducers and activators of transcription
PPARγ	peroxisome proliferator-activated receptor γ
SREBP	sterol regulatory element binding proteins
TG	triglycerides
HDL	high density lipoprotein
LDL	low density lipoprotein
CHO	total cholesterol
*FABP4*	fatty acid binding protein fatty 4
*FASn*	fatty acid synthetase
*Acox*	acyl CoA oxidase
NOAEL	no observed adverse effect level
IHC	immunohistochemistry.
